# Nitrite and nitrate in meat processing: Functions and alternatives

**DOI:** 10.1016/j.crfs.2023.100470

**Published:** 2023-02-24

**Authors:** Yin Zhang, Yingjie Zhang, Jianlin Jia, Haichuan Peng, Qin Qian, Zhongli Pan, Dayu Liu

**Affiliations:** a Meat Processing Key Laboratory of Sichuan Province, Chengdu University, Chengdu, 610106, China; b Department of Biological and Agricultural Engineering, University of California, Davis, One Shields Avenue, Davis, CA, 95616, USA

**Keywords:** Meat products, Meat processing, N-nitroso compounds, Meat safety, Red meat

## Abstract

Meat and meat products are important foods in the human diet, but there are concerns about their quality and safety. The discovery of carcinogenic and genotoxic N-nitroso compounds (NOCs) in processed meat products has had serious negative impacts on the meat industry. In order to clarify the relationship between the use of nitrite or nitrate and the safety of meat or meat products, we reviewed NOCs in meat and meat products, the origin and safety implications of NOCs, effects of nitrite and nitrate on meat quality, national regulations, recent publications concerning the using of nitrite and nitrate in meat or meat products, and reduction methods. By comparing and analyzing references, (1) we found antioxidant, flavor improvement and shelf-life extension effects were recently proposed functions of nitrite and nitrate on meat quality, (2) the multiple functions of nitrite and nitrate in meat and meat products couldn't be fully replaced by other food additives at present, (3) we observed that the residual nitrite in raw meat and fried meat products was not well monitored, (4) alternative additives seem to be the most successful methods of replacing nitrite in meat processing, currently. The health risks of consuming processed meat products should be further evaluated, and more effective methods or additives for replacing nitrite or nitrate are needed.

## Highlights

• Residual nitrite in raw meat products were not well monitored.
• Alternative additives seems the most successful to replace nitrite or nitrate.
• The multiple functions of nitrite cannot be completely replaced.
• More effective methods for replacing nitrite or nitrate are needed.

## Graphical abstract

**Figure:**
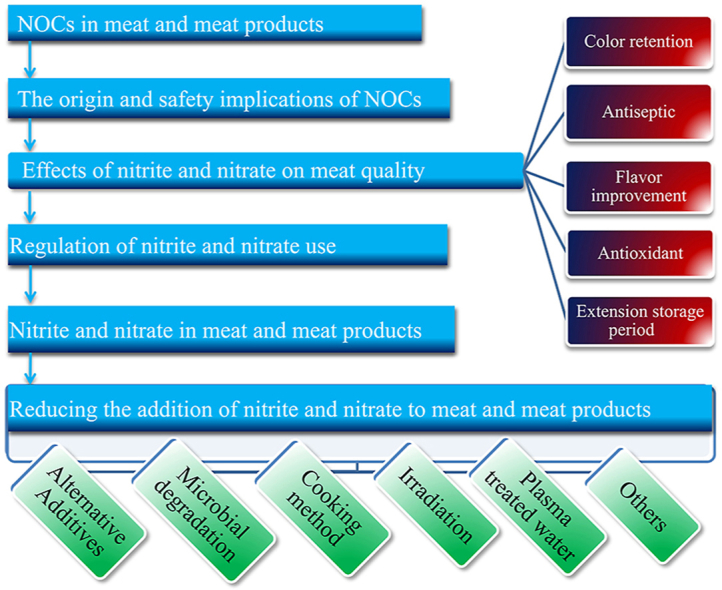

## 1 Introduction

Meat and meat products are important sources of energy and nutrients for humans. The important position of meat and meat products in human foods is shown by the long history of their consumption, which can be traced back to about 3 million years ago (Chris, 2020; Guo et al., 2005; McKenna, 2019). Since mankind's earliest days, animal meat has been one of our species' major dietary materials, enabling primitive man to sustain a living. However, fresh meat is not stable to be stored for a long term. It will deteriorate within several days. This problem was solved by primitive man about 1.5 million years ago, with the application of fire to meat processing. Grilled meat from animals killed by forest fires tasted better than fresh meat. Therefore, they experimented with fire for cooking meat, discovering that roasted or smoked fresh meat was more stable for storage (Zeng, 2007). Roasting and smoking thus became the earliest preservation methods for meat and meat products. Possibly because of the effectiveness of smoking for preserving meat, and the habit of eating meat with a smoky flavor, roasted or smoked meat products are still popular and widely consumed globally.

The actual reason for the current use of nitrate and nitrite salts in cured meat products is related to the ancient salting practices for meat preservation. Nitrate and nitrite were contaminant in salts used in salted meats. The advances in science allowed the discovery of nitrate (NO_3_^−^) first, and then nitrite (NO_2_^−^), as actual ingredients involved in cured meat products preservation (Bedale et al., 2016). NO_2_^−^ salts and NO_3_^−^ salts, such as those of sodium and potassium, are typical food preservatives used in meat processing. Both of them can inhibit the growth of microorganisms, delay the onset of rancidity, produce cured meat flavor or smell, and stabilize the meat's red color (Ferysiuk and Wojciak, 2020). The efficiency of nitrite and nitrate makes them indispensable additives for meat and meat products, especially for cured meat products (Ferysiuk and Wojciak, 2020). However, the discovery of N-nitroso compounds (NOCs) in the 1950s raised concerns about the safety of using nitrite or nitrate in meat processing (Bedale et al., 2016), which seriously threatened the healthy development of the global meat industry. In addition, with widely acceptance of the Clean label movement, having fewer ingredients become mainstream in food processing (Asioli et al., 2017). In order to clarify the relationship between the use of nitrite or nitrate and the safety of meat or meat products, this review focuses on NOCs in meat and meat products, the origin and safety implications of NOCs, effects of nitrite and nitrate on meat quality, national regulations, recent publications concerning the using of nitrite and nitrate in meat or meat products, and reduction methods.

## 2 NOCS in meat and meat products

**Fig. 1:**
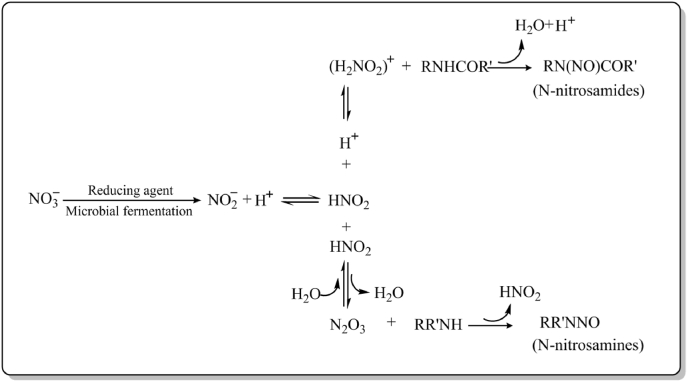
The proposed mechanism for the conversion of nitrite and nitrate to NOCs.

NOCs mainly consist of N-nitrosamines (RR'NNO) and N-nitrosamides (RN(NO)COR’). According to Dietrich et al. (2005) and Honikel (2008b), the proposed mechanism for the conversion of nitrite and nitrate to NOCs is shown in Fig. 1. RR'NNO and RN(NO)COR’ are formed by the nitrosation of amines and amides, respectively. *In vitro*, thiocyanates naturally produced in foods accelerate the nitrosation of amines, and organic acid food additives catalyze the nitrosation of amides. Some bacteria with nitrate-reducing or nitrosating activity can also promote the formation of NOCs, but the production of NOCs can be inhibited by adding vitamins C and E (Dietrich et al., 2005), the other methods of reducing the production of NOCs were enumerated at section 7 of this review.

A list of recently identified NOCs in meat and meat products is shown in Table 1. There are 14 volatile and 11 non-volatile types of NOC. Among the N-nitrosamines reported most frequently in meat products, N-Nitrosodimethylamine (NDMA), and N-nitrosomethylethylamine (NDEA) are considered the most dangerous in terms of carcinogenicity and genotoxicity (De Mey et al., 2017; Ferysiuk and Wojciak, 2020). However, recent studies have identified N-nitroso-diisopropylamine (NDIPA) in sausages and ham using a new gas chromatography-tandem mass spectrometry method (Yang et al., 2019). According to NDIPA's safety description, it is a group 1B carcinogenic chemical, and more toxic than NDMA and NDEA. This discovery suggests that there might be other undetected toxic NOCs in meat or meat products, and therefore, developing new methods to identify the compounds is very important for the timely discovery and monitoring of NOCs in meat or meat products.

**Table 1:** NOCs generated in meat and meat products.

Category	Substance	CAS no.	Carcinogenicity b	TDLo^a^ (dose/duration)	Genotoxicity^c^	DNA damage dose(μg/kg bw)	References
volatile NOCs	N-nitroso-diisopropylamine (NDIPA)	601-77-4	1B	14 mg/kg bw/110W–C	Genotoxic *in vitro* and *in vivo*	No data	(Mortensen et al., 2017; Yang et al., 2019)
	N-nitrosodimethylamine (NDMA)	62-75-9	2A	23 mg/kg bw/2Y–I	Genotoxic *in vitro* and *in vivo*	22	(FAOSTAT, 2021; Mortensen et al., 2017)
	N-nitrosomorpholine (NMOR)	59-89-2	2B	320 mg/kg bw	Genotoxic *in vitro* and *in vivo*	400	(Asioli et al., 2017; Mortensen et al., 2017)
	N-nitrosomethylethylamine (NMEA)	10595-95-6	2B	600 mg/kg bw/15W–I	Positive *in vitro* (DNA binding)	25	Mortensen et al. (2017)
	N-nitrosopyrrolidine (NPYR)	930-55-2	2B	476 mg/kg bw/3.3Y–C	Genotoxic *in vitro* and *in vivo*	5 × 10^c^	(FAOSTAT, 2021; Mortensen et al., 2017)
	N-nitrosodiethylamine (NDEA)	55-18-5	2A	119 mg/kg bw/3.3Y–C	Genotoxic *in vitro* and *in vivo*	67	(FAOSTAT, 2021; Mortensen et al., 2017)
	N-nitrosopiperidine (NPIP)	100-75-4	2B	350 mg/kg bw/2Y–I	Genotoxic *in vitro*	2220	Mortensen et al. (2017)
	N-nitroso-di-n-propylamine (NDPA)	621-64-7	2B	660 mg/kg bw/60W–I	Genotoxic *in vitro*	310	Mortensen et al. (2017)
	N-methyl-N-phenylnitrous amide(NMPHA)	614-00-6	No data	61 mg/kg bw/29W–C	No data	No data	(Asioli et al., 2017; Yang et al., 2019)
	N-ethyl-N-phenylnitrousamide(NEPHA)	612-64-6	No data	No data	No data	No data	Yang et al. (2019)
	N-nitrosodiphenylamine(NDPhA)	86-30-6	No data	140 mg/kg bw/2Y–C	No data	No data	Zhao et al. (2017b)
	N-nitrosodihexylamine(NDHA)	6949-28-6	No data	No data	No data	No data	Xing (2010)
	N-nitrosomethylaniline (NMA)	614-00-6	No data	61 mg/kg bw/29W–C	Limited positive data *in vitro* (Ames test)	No data	Mortensen et al. (2017)
Non-volatile NOCs	N-nitrosohydroxyproline (NHPRO)	30310-80-6	3	No data	No data	No data	Mortensen et al. (2017)
	N-nitrosoproline (NPRO)	7519-36-0	3	770 mg/kg bw/8W–I	Limited negative data (Ames test)	No data	Mortensen et al. (2017)
	N-nitrososarcosine (NSAR)	13256-22-9	2B	29 mg/kg bw/41W–C	Limited negative data (host-mediated assay)	No data	Mortensen et al. (2017)
	N-nitrosodibutylamine (NDBA)	924-16-3	2B	140 mg/kg bw/4W–C	Genotoxic *in vitro* and *in vivo* (Ames test, V79/hprt, *in vivo* comet and SCE)	83	Mortensen et al. (2017)
	N-nitrosodiisobutylamine (NDiBA)	997-95-5	No data	1750 mg/kg bw/30W–I	Limited positive data*in vitro*(Ames, V79/hprt)	No data	Mortensen et al. (2017)
	N-nitrosodibenzylamine (NDBzA)	5336-53-8	No data	No data	Limited positive data *in vitro* (Ames test)	5 × 10^d^	Mortensen et al. (2017)
	N-nitroso-2-hydroxymethyl-thiazolidine-4-carboxylic acid (NHMTCA)	99452-46-7	No data	No data	No data	No data	Mortensen et al. (2017)
	N-nitroso-thiazolidine-4-carboxylic acid (NTCA)	88381-44-6	No data	No data	No data	No data	Mortensen et al. (2017)
	N-nitroso-2-methyl-thiazolidine 4-carboxylic acid (NMTCA)	103659-08-1	No data	No data	No data	No data	Mortensen et al. (2017)
	N-nitrosopipecolic acid (NPIC)	4515-18-8	No data	No data	No data	No data	Mortensen et al. (2017)
	N-nitroso-N,N-di-(7-methyloctyl)amine(NDiNA)	643014-99-7	No data	No data	No data	No data	Ma et al. (2020)

41B represents the agent is carcinogenic to humans; 2A represents the agent is probably carcinogenic to humans; 2B represents the agent is possibly carcinogenic to humans; 3 represents the agent is not classifiable as to its carcinogenicity to humans.

a TDLo means lowest published toxic dose.

b Carcinogenicity date from IARC (The International Agency for Research on Cancer) group in monographs supplement 7, 1987 (http://www.iarc.fr/en/publications/list/monographs/index.php) and Globally Harmonized System of Classification and Labelling of Chemicals (https://www.sigmaaldrich.cn/CN/en/sds/supelco/y0002263).

c Genotoxicity of each NOCs was from hazardous substances Data Bank (http://toxnet.nlm.nih.gov/newtoxnet/hsdb.htm).

d Carcinogenicity TDLo and DNA damage dose of each NOCs was from Chemical Toxicity Database (https://www.drugfuture.com/toxic/search.aspx), the test system was rat, the route of exposure was oral.

## 3 The origin and safety implications of NOCS

The addition of nitrite to meat or meat products may induce the formation of NOCs (Ferysiuk and Wojciak, 2020). The World Cancer Research Fund stated that eating red or processed meat was likely to increase the risk of colorectal cancer (CRC) in 2007 (Rajkovic et al., 2017). Given the higher cancer risk associated with eating red and processed meat (Crowe et al., 2019), the IARC classified all types of mammalian muscle meat, such as beef, veal, pork, lamb, mutton, horse, and goat, as probably carcinogenic (Group 2A), and processed meat (treated with salting, curing, fermentation, smoking, or other processes to enhance flavor or improve preservation) as carcinogenic to humans (Group 1) (IARC, 2018).

Exposure to nitrite or nitrate may occur via many sources besides red meat and its related products. Table 2 indicates that consumption of vegetables is responsible for approximately 80% of nitrite exposure and 85% of nitrate exposure, while the consumption of meat and meat products accounts for only about 5% of nitrite exposure (Ferysiuk and Wojciak, 2020). Although these exposure ratios may be only approximate, they demonstrate that vegetables, nitrate reduction, and water are the three major sources of nitrite exposure in humans. Further investigation is needed into the contributions of consuming meat or meat products treated with nitrite to cancer risks in humans. In order to promote the healthy development of the meat industry and ensure human health, the purpose of this review is to evaluate the recent developments and current knowledge concerning nitrite and nitrate in meat processing and alternative methods to reduce the residual amount of nitrite and nitrate in meat or meat products.

**Table 2:** The nitrite and nitrate sources and their exposure status to human.

Sources	Ways to enter human body	Human exposure status	References
Vegetable and its products	Fresh vegetables, cooked vegetables, vegetable products, vegetable extracts	approximately 80% nitrite exposure, 85% nitrate exposure	(Karwowska and Kononiuk, 2020; Ranasinghe, 2018)
Oral reduction of nitrate	Nitrate-reducing bacteria can trandser about 5–7% of all ingested nitrate to nitrite at the base of the tongue	approximately 70–80% of the human total nitrite exposure	(Chan, 2011; Gassara et al., 2016; Leth et al., 2008)
Water	Drinking water, beverage, aquatic products, liquid condiments, agriculture products fertilized with natural organic wastes or overused fertilizers	approximately 5–40% dietary nitrate intake, if the nitrate level in drinking-water is more than 50 mg L^−1^, it wiil be the main source of exposure to nitrates	(Addiscott, 2005; Elias et al., 2020; Karwowska and Kononiuk, 2020)
Meat and meat products	Fresh meat, chilled meat, freezing meat, cured meat products, sauced and stewed meat, roasting meat burning smoke, dried meat, fried meat products, sausage meat products, prepared meat products, ham sausage, others	approximately 5% nitrite exposure, it depends the category of meat and meat products	(Colla et al., 2018; Ferysiuk and Wojciak, 2020; General Administration of Quality Supervision et al., 2011)
Fish and fish products	Fresh fish, chilled fish, freezing fish, fish products, overcooked fish	No data. It depends the amount of consumption	Iammarino et al. (2013)
Dairy products	Liquid milk, milk powder, other dairy products	No data. It depends on the amount of consumption.	Gangolii et al. (1994)
Fruit and its products	Fresh fruits, fruit juice beverage, canned fruit, dried fruit slices, jam, fruit powder, pickle fruit	No data. It depends on the amount of consumption.	(Ding et al., 2018; Ferysiuk and Wojciak, 2020)
Herbs	Herbal medicine, plant seasoning, functional foods, beverage,	No data. It depends on the amount of consumption.	Colla et al. (2018)
Cereals	Fertilizer, irrigation water	No data. It depends on the plant management.	(Karwowska and Kononiuk, 2020)
Medicine	Therapeutic medicine, patient, therapeutic treatments for angina and digital ischemia	No data. It depends on the patient's conditions.	(Cross et al., 2011; Karwowska and Kononiuk, 2020)
Food additives	L-arginine, decarboxylase and amino acid	No data. It depends on the addition amount and process.	(Flores and Toldra, 2021)

## 4 Functionality of nitrite and nitrate on meat quality

### 4.1 Color formation effect

Nitrite or nitrate can produce a pinkish-red color via interaction with the myoglobin in muscle. In addressing the food safety concerns, most researchers have, in recent years, focused on finding replacement color formation ingredients containing natural nitrite or nitrate, such as vegetable extracts and bacteria. Jarulertwattana et al. (2021) found that 4 × 10^−6^ g/g of nitrite was suitable for chicken meat marination when it was used with ginger paste, whereas more than 4 × 10^−6^ g/g of nitrite will produce pink color defect in the drumsticks. Zhu et al. (2020) used *Lactobacillus plantarum* to partially replace standard nitrite in pork sausages, finding that 50 mg/kg NaNO_2_ combined with 7 log CFU/g *L. plantarum* produced similar effects to 100 mg/kg NaNO_2_ alone. Ozaki et al. (2020) added radish powder (0.5% or 1.0%) and oregano essential oil (100 mg/kg) to fermented cooked pork and beef sausage, and found that both treatments could improve the red color of these products. Higuero et al. (2020) found that 75 mg/kg nitrate or 37.5 mg/kg nitrite produced a similar color to that seen following 150 mg/kg nitrate or 150 mg/kg nitrite treatment. This research suggests that, in terms of red color formation, there is an optimal amount of nitrite or nitrate. Therefore, appropriate process optimization may be an advisable method for controlling the amount of nitrite or nitrate added. Typical process optimization methods are orthogonal experiment design and response surface experimental design (Zhang et al., 2012). The latter, in particular, has been successfully used in the optimization of processes involving meat or other food products (Zhang et al., 2014, 2015, 2019, 2021; Zhao et al., 2017a). Recently, Kim and Chin (2021) evaluated the effects of paprika oleoresin, sunflower seed oil, and sodium nitrite on the storage quality of emulsified pork sausage, and the results indicated that this combination, given as 7.5 × 10^−5^ NaNO_2_+ 0.1% paprika oleoresin solution (1% paprika oleoresin +99% sunflower seed oil or 5% paprika oleoresin +95% sunflower seed oil) could increase redness.

### 4.2 Antimicrobial properties

The antimicrobial effects of nitrate and nitrite are considered very important in meat and meat products. The minimum concentration of nitrite that inhibits the outgrowth of *Clostridium botulinum* is 4 × 10^−5^ –8 × 10^−5^ g/g (Rivera et al., 2019). Patarata et al. (2020) investigated the effects of red wine and garlic on the behavior of *C. sporogenes* (used as a substitute for *C. botulinum*) and *Salmonella* in a dry-cured chouriço sausage, and found that red wine (7.5%) and red wine + garlic (7.5% + 1%, respectively) could destroy *Clostridium sporogenes* and *Salmonella* during the processing of chouriço. Khatib et al. (2020) used hop components to replace nitrite in the preparation of cooked beef sausage by means of lupulon–xanthohumol-loaded nanoliposomes (XLN) used in the production process; the results indicated that 1.5 × 10^−4^ XLN and 3 × 10^−5^ nitrite could produce bacteriostasis during 30 days of storage at 4 °C. Radish powder and oregano essential oil can produce nitrite in fermented cooked pork and beef sausages, thus inhibiting mesophilic bacteria (Ozaki et al., 2020). Kim and Chin (2021) found that the addition of paprika oleoresin, sunflower seed oil, and sodium nitrite (7.5 × 10^−5^ NaNO_2_ + 0.1% paprika oleoresin solution [1% paprika oleoresin +99% sunflower seed oil or 5% paprika oleoresin +95% sunflower seed oil]) could decrease the total plate counts of emulsified pork sausage samples. Vegetable extracts alone or in combination with other ingredients thus show promising abilities to control microorganisms in meat or meat products, but the specific chemicals that result in the antimicrobial effect are not well understood. Therefore, more investigations should be performed to identify the underlying processes responsible for the antimicrobial effect of vegetable extracts on meat or meat products.

### 4.3 Flavor improvement

The effect of microbial starters on the curing flavor of meat products has become a hot research topic. Perea-Sanz et al. (2020) investigated the effect of *Debaryomyces hansenii* inoculation on the aroma chemicals of dry fermented sausage and found that *D. hansenii* could induce the generation of potent aroma compounds such as ethyl ester and 3-methylbutanal. TIAN et al. (2020b) used *Lactobacillus fermentum* RC4 to decrease the nitrite level in salted meat, and the results indicated 73 volatile substances were identified in the fermented group, while there were 67 volatile substances in the control. Zhao (2020) investigated the effects of *lactobacillus helveticus* TR1-1-3 and ZF22 on the flavor chemicals of mutton sausages, and found that 1-pentene-3-ol, 1-octene-3-ol, and 3-hydroxy-2-butanone were typical flavor chemicals in fermented sausages. Huang et al. (2021) investigated the effects of *Lactobacillus* starter culture (*Lactobacillus fermentum* RC4, *L. plantarum* B6) on the volatile flavor elements in cured meat. The results indicated the key volatile components included D-limonene and nonanal, (E,E)-2, 4-decadienal, 1-octene-3-ol, and anethole. Nitrite can inhibit the growth of bacteria like *Clostridium botulinum*, *Bacillus cereus*, *Staphylococcus aureus*, *Clostridium perfringens*, and others (Dong and Tu, 2006). This effect of nitrite can assist some fermentative bacteria in cured meat products to produce fermented flavor ingredient by the metabolism of themselves or the hydrolysis of proteases and lipases in them (Chen et al., 2021; Wang et al., 2021). Therefore, nitrite may indirectly affect development of cured meat flavor via its impact on the activity of microorganisms and endogenous enzymes.

### 4.4 Antioxidant effect

The NO engendered by nitrite can competitively deplete oxygen by self-oxidation, bind to the iron ion in hemoglobin and thus prevent its oxidization, and destroy the radical chain reactions of lipid oxidations (Jo et al., 2020). These indirect effects of nitrite may explain the antioxidant effects of nitrite. Ji et al. (2020b) found that sodium nitrite can effectively inhibit lipid oxidation at 1 × 10^−4^ in mutton marinating process. Khatib et al. (2020) used L–X-NL (lupulon–xanthohumol loaded nanoliposome) to replace nitrite in the preparation of cooked beef sausage, and found that these nanoliposomes could prevent lipid oxidation. Ma et al. (2021) compared the antioxidant effects of phosphorylated nitrosohemoglobin (PNHb) and sodium nitrite on emulsified sausage; the results indicated that the thiobarbituric acid values of the PNHb group and NaNO_2_ group were 0.62 mg/kg and 0.67 mg/kg, respectively, suggesting that PNHb has a stronger antioxidant effect than NaNO_2_. Despite the results, nitrite is still widely accepted as a color formation and antiseptic agent in most countries.

### 4.5 Shelf-life extension

The improvement in shelf life is actually a combination of many factors delaying the loss of quality, including the hygiene of raw meat, processing treatments, storage temperature and relative humidity, packaging method, etc (Singh et al., 2011). For nitrite and nitrate, they can extend the shelf-life of meat and meat products by inhibiting the outgrowth of pathogenic and spoilage bacteria (Rivera et al., 2019). Recently, Chatkitanan and Harnkarnsujarit (2020) combined thermoplastic starch, sodium nitrite, and low-density polyethylene to develop a starch-based, nitrite-containing film. They used it to vacuum-package pork, and the results indicated that the film not only increased the redness of pork by more than 30% during chilled storage, but also inhibited microbial growth and lipid oxidation. Khatib et al. (2020) found that the addition of XLN to cooked beef sausage could extend its storage life via bacteriostasis and prevent fat oxidation during 30 days of storage at 4 °C.

## 5 Regulation of nitrite and nitrate use

The multiple functions of nitrite and nitrate in meat and meat products cannot be fully replaced by other food additives at present (Karwowska and Kononiuk, 2020). Complete restriction of their use in this context may not be acceptable to producers and consumers. In order to regulate the application of nitrite and nitrate in meat or meat products, many countries have established directives and regulations (Table 3) (Gassara et al., 2016).

**Table 3:** Addition limit of nitrite and nitrate in meat and meat products in different countries.

Country or organization	Meat and meat products	Limit of Nitrite	Limit of Nitrate	References
The United Nations	Cured ham and cooked cured pork shoulder	Residual nitrite level≤8 × 10^−5^	/	(Additives and C. C. C. o. F., 2015)
	Heat-treated processed meat, poultry, and game products in whole pieces or cuts	Residual nitrite level≤8 × 10^−5^	/	(Additives and C. C. C. o. F., 2015)
	Processed comminuted meat, poultry, and game products	Residual nitrite level≤8 × 10^−5^	/	(Additives and C. C. C. o. F., 2015)
	Luncheon meat, cooked cured chopped meat	Residual nitrite level≤8 × 10^−5^	/	(Additives and C. C. C. o. F., 2015)
	corned beef	Residual nitrite level ≤3 × 10^−5^	/	(Additives and C. C. C. o. F., 2015)
The United States	Pumped and/or massaged bacon	The ingoing level of sodium nitrite ≤1.2 × 10^−4^ (or 1.48 × 10^−4^ potassium nitrite)	/	(USDA and Agriculture, 1995)
	Iimmersion-cured bacon	The ingoing level of sodium nitrite ≤1.2 × 10^−4^ (or 1.48 × 10^−4^ potassium nitrite)	/	(USDA and Agriculture, 1995)
	Comminuted meat and poultry products	The ingoing level of sodium (or potassium) nitrite≤1.56 × 10^−4^	/	(USDA and Agriculture, 1995)
	Massaged or pumped meat and poultry products	The ingoing level of sodium (or potassium) nitrite≤2 × 10^−4^	/	(USDA and Agriculture, 1995)
	Immersion cured meat and poultry products	The ingoing level of sodium (or potassium) nitrite≤2 × 10^−4^	/	(USDA and Agriculture, 1995)
	Dry cured bacon	The ingoing level of sodium (or potassium) ≤2 × 10^−4^ (or 2.46 × 10^−4^ potassium nitrite)	/	(USDA and Agriculture, 1995)
	Dry cured meat and poultry products	The ingoing level of sodium (or potassium) nitrite≤6.25 × 10^−4^	/	(USDA and Agriculture, 1995)U
European union	Canned meat products	The ingoing level of nitrite≤1.5 × 10^−4^	/	Liu et al. (2019)
	Meat products	The ingoing level of potassium nitrite≤1.5 × 10^−4^ (expressed as NaNO_2_)	/	Wei et al. (2009)
	Cured meat products	/	The ingoing level of sodium or potassium nitrate ≤3 × 10^−4^; Residual amounts (sodium or potassium nitrate)≤2.5 × 10^−4^	Honikel (2008a)
	Other cured meat products	The ingoing level of sodium nitrite ≤1.5 × 10^−4^; Residual amounts (Sodium nitrite)≤1 × 10^−4^	/	Honikel (2008a)
	Cured bacon	The ingoing level of sodium nitrite ≤1.5 × 10^−4^; Residual amounts (Sodium nitrite)≤1.75 × 10^−4^	/	Honikel (2008a)
	Dry cured bacon	Residual amounts (nitrites and nitrates) 4.25 × 10^−4^	/	Gassara et al. (2016)
	Non-heat-treated processed meat	Maximum added amount (sodium or potassium nitrite) during manufacturing (expressed as NaNO_2_) ≤1.5 × 10^−4^ Residual amounts (Potassium nitrite)≤5 × 10^−5^	Maximum added sodium or potassium nitrate amount ((expressed as NaNO_2_)) during manufacturing ≤1.5 × 10^−4^	(Ahn et al., 2002; Honikel, 2008a)
	Heat-treated processed meat, except sterilised meat products (3 min heating at 121 °C for C. botulinum)	Maximum added amount (sodium or potassium nitrite) during manufacturing ≤1.5 × 10^−4^	/	Ahn et al. (2002)
	Sterilised meat products(3 min heating at 121 °C for C. botulinum)	The ingoing level of sodium nitrite ≤1 × 10^−4^ (expressed as NaNO_2_)	/	Wei et al. (2009)
	Other traditionally cured meat products (number of products)	Maximum amount of sodium nitrite that may be added during manufacturing (expressed as NaNO_2_) ≤1.8 × 10^−4^; Maximum residual sodium nitrite level (expressed as NaNO_2_) ≤5 × 10^−5^	Maximum sodium nitrate amount that may be added during manufacturing (expressed as NaNO_2_) is 2.5 × 10^−4^-3 × 10^−4^ (without nitrite added) Residual sodium nitrate amounts (expressed as NaNO_2_) 1 × 10^−5^-2.5 × 10^−4^	(Honikel, 2008a; Wei et al., 2009)
	Only sterilised meat products (3 min heating at 121 °C for C. botulinum)	Maximum added amount (sodium or potassium nitrite) during manufacturing ≤1 × 10^−4^	/	Ahn et al. (2002)
	Traditional immersion cured meat products (number or products)	Maximum residual sodium nitrite level (expressed as NaNO_2_) 5 × 10^−5^–1.75 × 10^−4^	Maximum added sodium nitrate amount (expressed as NaNO_2_) during manufacturing ≤3 × 10^−4^; Residual sodium nitrate amounts (expressed as NaNO_2_) is 1 × 10^−5^–2.5 × 10^−4^ (some without added)	Wei et al. (2009)
	Traditional dry cured meat products (number of products)	Maximum residual sodium nitrite level (expressed as NaNO_2_) is 5 × 10^−5^–1.75 × 10^−4^	Maximum added sodium nitrate amount (expressed as NaNO_2_) during manufacturing ≤3 × 10^−4^; Residual sodium nitrate amounts (expressed as NaNO_2_) is ≤ 5 × 10^−5^ (some without added)	Wei et al. (2009)
China	Cured meat products (such as bacon, cured meat, salted duck, Chinese ham and sausage)	Maximum addition amount (sodium (potassium) nitrite) ≤ 0.5 g/kg; Based on sodium nitrite, the residue is ≤ 3 × 10^−5^	Maximum addition amount (sodium (potassium) nitrate) ≤ 0.5 g/kg; Based on sodium (potassium) nitrite, the residue is ≤ 3 × 10^−5^	National Health and Family Planning Commission (2015)
	sauced and stewed meat products	Maximum addition amount (sodium (potassium) nitrite) ≤ 0.5 g/kg; Based on sodium nitrite, the residue is ≤ 3 × 10^−5^	Maximum addition amount (sodium (potassium) nitrate) ≤ 0.5 g/kg; Based on sodium (potassium) nitrite, the residue is ≤ 3 × 10^−5^	National Health and Family Planning Commission (2015)
	Smoked and roasted meat products	Maximum addition amount (sodium (potassium) nitrite) ≤ 0.5 g/kg; Based on sodium nitrite, the residue is ≤ 3 × 10^−5^	Maximum addition amount (sodium (potassium) nitrate) ≤ 0.5 g/kg; Based on sodium (potassium) nitrite, the residue is ≤ 3 × 10^−5^	National Health and Family Planning Commission (2015)
	Fried meat products	Maximum addition amount (sodium (potassium) nitrite) ≤ 0.5 g/kg; Based on sodium nitrite, the residue is ≤ 3 × 10^−5^	Maximum addition amount (sodium (potassium) nitrate) ≤ 0.5 g/kg; Based on sodium (potassium) nitrite, the residue is ≤ 3 × 10^−5^	National Health and Family Planning Commission (2015)
	Western style ham (smoked and roasted, smoked, stewed)	Maximum addition amount (sodium (potassium) nitrite) ≤ 0.5 g/kg; Based on sodium nitrite, the residue is ≤ 7 × 10^−5^	Maximum addition amount (sodium (potassium) nitrate) ≤ 0.5 g/kg; Based on sodium (potassium) nitrite, the residue is ≤ 3 × 10^−5^	National Health and Family Planning Commission (2015)
	Sausage meat products	Maximum addition amount (sodium (potassium) nitrite) ≤ 0.5 g/kg; Based on sodium nitrite, the residue is ≤ 3 × 10^−5^	Maximum addition amount (sodium (potassium) nitrate) ≤ 0.5 g/kg; Based on sodium (potassium) nitrite, the residue is ≤ 3 × 10^−5^	National Health and Family Planning Commission (2015)
	Fermented meat products	Maximum addition amount (sodium (potassium) nitrite) ≤ 0.5 g/kg; Based on sodium nitrite, the residue is ≤ 3 × 10^−5^	Maximum addition amount (sodium (potassium) nitrate) ≤ 0.5 g/kg; Based on sodium (potassium) nitrite, the residue is ≤ 3 × 10^−5^	National Health and Family Planning Commission (2015)
	Canned meat products	Maximum addition amount (sodium (potassium) nitrite) ≤ 0.5 g/kg; Based on sodium nitrite, the residue is ≤ 5 × 10^−5^		National Health and Family Planning Commission (2015)
Canada	Cured meat and meat by-products (except bacon)	Residual levels of nitrites ≤2 × 10^−4^	Residual levels of nitrate ≤2 × 10^−4^	Gassara et al. (2016)
	bacon	Residual levels of nitrites ≤1 × 10^−4^	Residual levels of nitrate ≤1 × 10^−4^	Gassara et al. (2016)
Korean	Meat products, meat extract processed products, edible beef tallow, and edible pork	Residual nitrite level <7 × 10^−5^	/	Safety (2013)
	Fish sausages	Residual nitrite level <5 × 10^−5^	/	Safety (2013)
	Salted pollack roe and salmon roe	Residual nitrite level <5 × 10^−6^	/	Safety (2013)
Japan	Meat products	Residual nitrite level <7 × 10^−5^	/	Cui et al. (2010)
India	Fermented nonheated treated processed meat and poultry products in whole pieces or cuts	Maximum level≤8 × 10^−5^	/	Government (2011)
	Heat-treated processed meat and poultryproducts in whole pieces or cuts (canned chicken, canned mutton and goat meat)	Maximum level≤8 × 10^−5^	/	(FSSAI), 2011)
	Processed comminuted meat and poultry products	Maximum level≤8 × 10^−5^	/	(FSSAI), 2011)
Mexico	Cooked meat product	Maximum addition≤1.56 × 10^−4^	Maximum addition≤1.56 × 10^−4^	Government (2012)
	Raw cured meat product	Maximum addition≤1.56 × 10^−4^	Maximum addition≤1.56 × 10^−4^	Government (2012)
	Cured and matured meat product	Maximum addition≤1.56 × 10^−4^	Maximum addition≤1.56 × 10^−4^	Government (2012)
	Smoked fishery products	Maximum addition≤1.56 × 10^−4^	Maximum addition≤1.56 × 10^−4^	Government (2012)
	Emulsified fishery products	Maximum addition≤1.5 × 10^−4^	Maximum addition≤1.5 × 10^−4^	Government (2012)
Argentina	Cured, mixed and semi-cured meat, Sausages: fresh or not dried, aged and/or not matured; Slaughter: fresh, cold or not dried, ripe or unripe; cooked or uncooked sausages	Residual sodium nitrite level≤1.5 × 10^−4^	Residual sodium nitrite level≤3 × 10^−4^	Salud (2021)
Brazil	Meat products	Maximum residual nitrite is 1.5 × 10^−4^	Maximum amount of NaNO_3_ and KNO_3_ that may be added during manufacturing 3 × 10^−4^	Della Betta et al. (2014) Della Betta et al. (2016)
Turkish	Meat products	Maximum residual nitrite ions ≤5 × 10^−5^	Maximum residual nitrate and ions ≤2.5 × 10^−4^	Büyükünal et al. (2016)
Sudan	Meat products	Maximum residual nitrite ions ≤1 × 10^−4^	/	Adam et al. (2016)
Denmark	Meat products	Maximum amount of NaNO_2_ that may be added during manufacturing ≤6 × 10^−5^	/	(Ferysiuk and Wojciak, 2020; Ministerium:-Miljø-og-Fødevareministeriet, 2018)

**Fig. 2:**
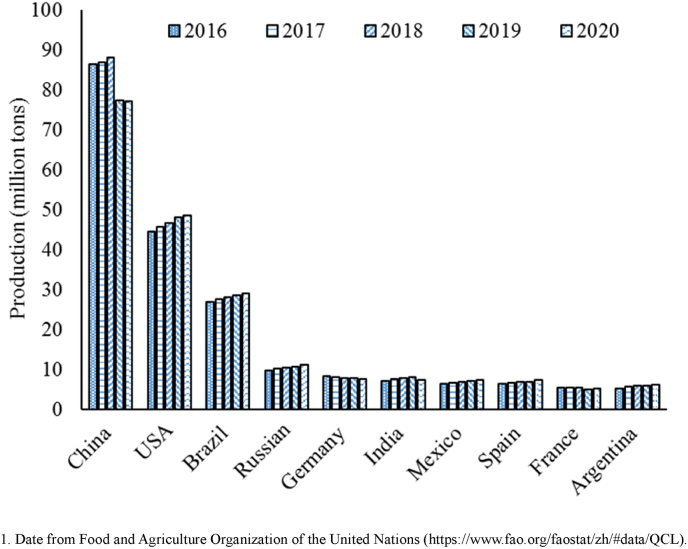
Top 10 countries in meat production quantity.

Germany is the fourth largest meat producer in the world (Fig. 2), and cured meat products are popular there. It was the first to propose limits to the addition of nitrite to meat and meat products, restricting the nitrite content in curing salt to between 0.5% and 0.6% (Ferysiuk and Wojciak, 2020). The United Nations only limits residual nitrite levels according to the category of meat product (Table 3). The United States is the second largest meat producer globally (Fig. 2), and its cured meat and meat products are frequently consumed as hotdogs or other convenience foods (Table 3). The maximum nitrite level for pumped and/or massaged bacon (the ingoing level of sodium nitrite ≤120 (or 148 ppm potassium nitrite) ppm) and other meat products were restrictedly limited (Table 3).

More specific restrictions have been imposed by the European Union and European countries (Table 3). They have restricted the levels of nitrite and nitrate in treatments and the residue levels in different meat products (Table 3). China is the world's largest meat producer (Fig. 2), cured meat products being widely-consumed traditional foods. To regulate the application of nitrite and nitrate in meat or meat products, similar limits have been proposed within Chinese additive standards (Table 3) (National Health and Family Planning Commission, 2015). Canada, Korea, Japan and other countries are all restricting input and residue levels of nitrite and nitrate in meat or meat products (Table 3).

Restricting input and residue levels is beneficial, in that it prevents the overuse of nitrite and nitrate in meat or meat products, but it is also essential to ensure that directives or regulations are implemented appropriately. Although most countries have established food inspection agencies to supervise the use of nitrite and nitrate in meat or meat products, there will be some omissions. Abd Hamid et al. (2020) investigated the nitrite intake of school children in Brunei Darussalam, and found that more than 20% of children exceeded the Acceptable Daily Intake (ADI) of nitrite (<0.07 mg/kg b. w/day). Therefore, frequent market sampling inspections are necessary to ensure the implementation of directives or regulations concerning nitrite or nitrate levels.

## 6 Nitrite and nitrate in meat and meat products

Meat production is an important index within global agriculture. The top ten meat-producing countries from 2015 to 2019, according to the Food and Agriculture Organization of the United Nations (FAO), are shown in Fig. 2. China and the USA are the two largest meat producers, jointly accounting for more than 60% of total world output. As such, their classification systems and data for meat products are the most detailed, and are reviewed below. These countries categorize meat and meat products into 11 types.

Two categories cover meat: raw meat and cooked meat; the remaining nine categories cover processed meat products (Table 4). Based on the categories listed in Table 4, the residual nitrite and nitrate in sausages (12) and ham (7) are the most frequently reported, while other types of meat products, in decreasing order of frequency, are roasted and burning smoked meat products (5), raw meat (4), cured meat products (4), dried meat products (2), other meat products (2), and sauced and stewed meat products (1). Few reports have been published concerning the residual nitrate or nitrite in fried meat products. The residual levels of nitrite and nitrate in sausage were 0–1.39 × 10^−4^ and 1.88 × 10^−4^ or less, respectively (Table 4). The residual levels of nitrite and nitrate in ham were 5.76 × 10^−5^ and 1.94 × 10^−4^ or less, respectively (Table 4). According to the European Commission's former Scientific Committee for Food and the Joint FAO/WHO Expert Committee on Food Additives, the ADIs for nitrite and nitrate are important for the health of consumers (Karwowska and Kononiuk, 2020). The recommended ADI for nitrite is less than 0.06 mg/kg bw/day; that for nitrate is 3.7 mg/kg bw/day (Santamaria, 2006). Taking the maximum amounts of nitrite (1.39 × 10^−4^) and nitrate (1.88 × 10^−4^) in sausage as examples, if an adult weighing 60 kg consumes 60 g of sausage, their nitrate intake will be below its ADI (0.188 mg/kg bw/day <3.7 mg/kg bw/day), but their nitrite intake will be above the daily acceptable level (0.139 mg/kg bw/day >0.06 mg/kg bw/day). Therefore, stricter application of regulatory restrictions and more frequent sampling surveys are required in countries where the ADIs for nitrite and nitrate are greater than 0.06 and 3.7 mg/kg bw/day, respectively.

**Table 4:** Recent investigations of nitrates or nitrites in meat or meant products.

Category	Area	Mean nitrite (mg kg^−1^)	Mean nitrate (mg kg^−1^)	References
Raw meat	Khartoum, Sudan	Minced meat 42	**/**	Adam et al. (2016)
	Iran	Beef 38.7	Beef 83.5	Bahadoran et al. (2016)
		Lamb 49.6	Lamb 74.3	
		Ground mixed meat 37.2	Ground mixed meat 124	
		Chicken meat 41.8	Chicken meat 133	
		Fish 33.9	Fish 55.6	
	Finland	Poultry 22.4	/	Suomi et al. (2016)
		Reindeer 12.0	/	
	Sydney/Australian	Minced beef 0	Minced beef 18.7	Hsu et al. (2009)
		Beef medallion 0	Beef medallion 38.5	
Cooked meat	Henan/China	Boiled beef 63. 91	**/**	Liang et al. (2016)
Cured meat products	Turkey	Pastırma 4.26–46.28	Pastırma 64.12–187.66	Büyükünal et al. (2016)
	Chengdu/China	cured meat products 4.0–8.7	/	Ji et al. (2020a)
	Philippines	Dumingag Chorizo 0.76–1.02	/	(Neri and Patalinghug, 2021)
		Molave Chorizo 0.15–1.73		
		Pagadian Chorizo 4.59–39.07		
		Molave Longaniza 0.55–2.54		
		Pagadian Longaniza 3.40–32.8		
	USA	Meat products 0.64–7.31	Meat products 14.81–78.81	Keeton et al. (2009)
Sauced and stewed meat products	Finland	Marinated pork 11.0	/	Suomi et al. (2016)
Roasted and burning smoked meat products	Korea	Bacon <31.3	/	(Choi et al., 2016
	Finland	Bacon 11.8	/	Suomi et al. (2016)
	Italian	Bacon 7.7	Bacon 178	Roila et al. (2018)
	South Korea	Bacon 15.8	/	(Institute), 2015)
	Sydney/Australians	Bacon 15.7	Bacon 23.3	Hsu et al. (2009)
Dried meat products	Korea	Dried meats <37.0	/	(Chen et al., 2016)
	Italian	Bresaola 25.67	Bresaola 188	Roila et al. (2018)
		Capocollo 0.76	Capocollo 69	
		Guanciale 8	Guanciale 142	
Sausage	Khartoum, Sudan	Sausage 51.8	/	Adam et al. (2016)
		Mortadella 28	/	
	Poland	Traditional sausages 5.79–14.78	Traditional sausages 12.51–34.64	Halagarda et al. (2018)
		Conventional sausages 9.72–79.21	Conventional sausages 13.79–27.80	
	Iran	Sausages 139	Sausages 188	Bahadoran et al. (2016)
	Korea	Sausage <55.1	/	(Choi et al., 2016)
	South Korea	Sausage 4.6	/	(Institute), 2015)
	Finland	Five types of sausages 8–97.6	/	Suomi et al. (2016)
	Brunei	Meat products 14.0	Meat products 20.4	Abd Hamid et al. (2020)
	Korea	/	Meat products 31.8	Chae et al. (2017)
	Turkey	Sucuk 6.41–90.02	Sucuk 28.10–174.62	Büyükünal et al. (2016)
	Sydney/Australian	Salami 0	142	Hsu et al. (2009)
		Frankfurt 83.9	Frankfurt 54.9	
	Italian	"Ciauscolo" salami 0	"Ciauscolo" salami 43	Roila et al. (2018)
		Dry fermented salami “Salame” 7.8	Dry fermented salami “Salame” 69	
		Dry fermented sausage “Salsiccia” 5.03	Dry fermented sausage “Salsiccia” 46	
	Tartu, Estonia	Frankfurters 20.2	Frankfurters 27.1	Elias et al. (2020)
		Dinner sausages 22.7	Dinner sausages 25.4	
		Boiled sausages 29	Boiled sausages 18.1	
		Other boiled sausages 25.8	Other boiled sausages 26.7	
		Semi-smoked sausages 18.9	Semi-smoked sausages 28.7	
		Fully smoked sausages 14.4	Fully smoked sausages 37.2	
		Salami type sausages 30.3	Salami type sausages 45.3	
		Liver pâté and pâté 26.7	Liver pâté and pâté 20.8	
		Uncooked raw sausages 17.8	Uncooked raw sausages 21.1	
Ham	Iran	Ham 57.6	Ham 194	Bahadoran et al. (2016)
	Italian	Cured ham 0	Cured ham 21	Roila et al. (2018)
	Australian	Ham 34.2	Ham 19.0	Hsu et al. (2009)
	Tartu, Estonia	Hams 22.8	Hams 12.7	(!!! INVALID CITATION !!!)
	South Korea	Ham 16.6	/	(Institute), 2015)
	Korea	Ham <57.4	/	(Choi et al., 2016)
	Finland	Ham 15.1	/	Suomi et al. (2016)
		Marinated pork 11.0	/	
		Prosciutto <8	/	
Prepared meat products	Khartoum, Sudan	Meat ball 51.9	/	Adam et al. (2016)
	Tartu, Estonia	Meat patties and meatballs 9.7	Meat patties and meatballs 10.8	Elias et al. (2020)
	Brunei	Meatballs 14.0	Meatballs 25.9	Abd Hamid et al. (2020)
	Korea	Meatballs 0	Meatballs 38.0	Chae et al. (2017)
	Iran	Meatballs 37.2	Meatballs 124.0	Bahadoran et al. (2016)
	Brunei	Meatloaves 14.0	Meatloaves 25.9	Abd Hamid et al. (2020)
Fried meat products	/	/	/	/
Others	Iran	Canned fish 29.3	Canned fish 60.9	Bahadoran et al. (2016)
	Egypt	Meat products 23–120	Meat products 55–200	Abdel-Moemin (2016)

To avoid indiscriminate use of nitrite and nitrate in meat or meat products, the scope for their addition is rigorously restricted in most countries. However, the data in Table 4 indicate that nitrite or nitrate have been added to raw meat materials, such as minced meat, beef, lamb, chicken meat, and fish. As ingredient or hazardous compound levels are not monitored by regulation in raw meat materials in some countries, and considering that nitrite overuse in processed meat products has occurred (Abd Hamid et al., 2020; Neri and Patalinghug, 2021), the application of nitrite or nitrate in raw meat materials may be a supervisory blind spot that increases the risk of overuse. Therefore, it is vital to strengthen the sampling regime for meat and meat products, especially raw meat where it is not currently included.

Fried meat products comprise one of the most widely consumed meat categories, but there are not many reports on their residual levels of nitrite or nitrate (Table 4). This may be because fried meat products are prepared meat products, which are readily consumed mixed with other foods, and usually eaten immediately after cooking. These features make them popular among adults or children and they are widely consumed in snack bars and homes. An investigation of nitrite intake in school children in Brunei indicated that more than 20% exceeded their nitrite ADI (0.06 mg/kg bw/day) by eating cured meat products (Abd Hamid et al., 2020). Therefore, in order to evaluate the safety hazards of eating fried meat products, more surveys of the residual nitrite or nitrate in fried meat products should be conducted, especially in products consumed by infants or children.

## 7 Reducing the addition of nitrite and nitrate to meat and meat products

### 7.1 Alternative additives

Following the classification of nitrite-cured meat as a Group 1 carcinogen by IARC (IARC, 2018), many researchers have tried to find alternative additives to replace nitrite or nitrate. Vitamins, vegetable extracts, spices, herbs, and fruits have all been investigated as alternatives (Flores and Toldra, 2021; Gassara et al., 2016), as these materials or their ingredients had shown antioxidant and/or bacteriostatic properties, preventing the formation of nitrosamines. Bamboo leaf extracts were found to prevent nitrite from transforming into N-nitrosamines by Pan et al. (2019), who added it to pork ham; the addition of 0.2% bamboo leaf extract could effectively stop the transformation of nitrite to N-nitrosamines. Wang et al. (2020) used rose extract to substitute for nitrite in the preparation of semi-dried fermented sausage, the resulting sausage quality was significantly improved over that of the sausage with 150 mg/kg sodium nitrite; the optimal combination was 10% rose extract and 80 mg/kg sodium nitrite. Khatib et al. (2020) prepared XLN and used them to replace nitrite in the preparation of cooked beef sausage, finding that this treatment can partially replace nitrite (50%) in the preparation of cooked beef sausage without impairing its sensory quality. Ozaki et al. (2020) added radish powder (0.5% or 1.0%) and oregano essential oil (100 mg/kg) to fermented cooked pork and beef sausages, and found that this mixture could improve their color and inhibit mesophilic bacteria, but it could not prevent lipid oxidation. Alirezalu et al. (2020) mixed plant extracts (stinging nettle, olive leaves, and green tea) with nisin or nisin nanoparticles, and compared their effects on color, fat oxidation, and the microorganism content of frankfurter sausages, finding that the mixture of plant extracts and nisin nanoparticles could prevent fat oxidation and inhibit the increase of bacteria, molds, and yeasts. They considered that the use of 2 × 10^−4^ g/g nisin nanoparticles and 5 × 10^−4^ g/g of mixed plant extract could enable production of nitrite-free frankfurter sausages of good quality with a stable shelf life. Recently, the addition of 25% guava epicarp flour extract in the production of frankfurters (Velasco-Arango et al., 2021), 0.2% *Flos Sophorae* and 1% *chili* pepper in Chinese sausages(Tang et al., 2021), 0.3% white kimchi powder and 0.5% lemon extract powder in naturally cured sausages (Bae et al., 2021), extracts of wild thyme by-product and potassium bixinate were all found effective to replace nitrite (Sojic et al., 2022). Other extracts from cruciferous vegetables as sources of nitrate in meat were reviewed by (Munekata et al., 2021).

### 7.2 Microbial degradation

Beyond the addition of vegetable juice or extracts to reduce the residual nitrite in meat or meat products, some microbes also showed nitrite reduction and conversion effects (Table 5). Microbial enzymes can convert metmyoglobin to NO-Mb, thereby making the cured meat pink. Huang et al. (2019) added *Lactobacillus fermentum* RC4 and *L. plantarum* B6, without any nitrite, during preparation of cured meat, producing a bright color and low nitrite content. Huang et al. (2020b) adopted three *staphylococcal* species (*S. vitulinus*, *S. carnosus*, and *S. equorum*) to induce the production of NO-Mb through nitric oxide synthase catalysis, which, in all three species, increased the a* value of the dry sausage model. Zhang et al. (2020b) inoculated *Lactobacillus curvatus* LAB26 and *Pediococcus pentosaceus* SWU73571, isolated from the sour meat of the Dong minority in China, into sour meat. Both bacteria decreased the nitrite content of sour meat and increased the a* value significantly. Luo et al. (2020) added *Lactobacillus fermentum* AS1.1880 into pork batters and confirmed that the species increased their redness by producing nitric oxide synthase. Tian et al. (2020a) found that *Lactobacillus fermentum* RC4 significantly reduced the nitrite content of salted meat. They combined *Lactobacillus fermentum* RC4 and *L. plantarum* B6 with beet red, Monascus red, and nisin to produce Chinese bacon, and found that the combined agents not only improved the cured meat quality, but also decreased the nitrite content. Liu et al. (2021) used the commercial starter PRO-MIX5 (*Staphylococcus xylose*, *Lactobacillus sakei*, *Lactobacillus plantarum*) to prepare bacon and found that the starter resulted in significantly lower NDMA and total N-nitrosamine levels.

**Table 5:** Major bacterial species that demonstrate nitrite reduction effect.

Microorganisms	Source of microorganisms	References
*Lactobacillus plantarum CMRC6*	Fermented pork sausage	Chen et al. (2016)
*Lactobacillus plantarum CMRC 3*	Guizhou Fermented Meat （Nanx Wudl）	Chen et al. (2018)
*Lactobacillus plantarum CMRC 19*	Guizhou Fermented Meat （Nanx Wudl）	Chen et al. (2018)
*Lactobacillus plantarum RC4*	Cured meat	Huang et al. (2019)
*Lactobacillus plantarum B6*	Cured prok sausage	Huang et al. (2020a)
*Lactobacillus fermentum*	Smoked fermented sausages; Chinese style sausage; Harbin red sausage	(Alahakoon et al., 2015; MoLler et al., 2003; Xue et al., 2007; Zhang et al., 2007)
*Lactobacillus fermentum RC4*	Cured prok sausage; salted meat	(Huang et al., 2020a; Tian et al., 2020a)
*Lactobacillus fermentum AS1.1880*	Fermented pork meat	Luo et al. (2020)
*Lactobacillus fermentum JCM1173 (generated nitric oxide myoglobin)*	Horse heart myoglobin	Arihara et al. (2010)
*Kurthia* sp. *K-22 (converted metmyoglobin to more desirable color derivatives)*	Horse heart myoglobin	Arihara et al. (2010)
*Chromobacterium violaceum K-28 (converted metmyoglobin to more desirable color derivatives)*	Horse heart myoglobin	Arihara et al. (2010)
*Lactobacillus curvatus LAB26*	Sour meat	Zhang et al. (2020b)
*Lactobacillus sakei CMRC15*	Fermented pork sausage	Chen et al. (2016)
*Staphylococcus vitulinus*	Dry sausage model	Huang et al. (2020b)
*Staphylococcus .equorum*	Dry sausage model	Huang et al. (2020b)
*Staphylococcus xylosus*	Raw pork meat batters; broth medium, raw meat batters	Li et al. (2013)
*Staphylococcus xylosus*	Raw pork meat batters; broth medium, raw meat batters	(Alahakoon et al., 2015; Li et al., 2013; Morita et al., 2010)
*Staphylococcus carnosus*	Sausages	(Alahakoon et al., 2015; Gotterup et al., 2008a)
*Staphylococcus saprophyticus*	Sausages	(Alahakoon et al., 2015; Gotterup et al., 2008a)
*Staphylococcus simulans*	Sausages	(Alahakoon et al., 2015; Gotterup et al., 2008a)
*Staphylococcus .carnosus*	Dry sausage model	Huang et al. (2020b)
*Staphylococcus strains (S. simulans, S. carnosus* subsp*.carnosus)*	Fermented pork sausage	Gotterup et al. (2008b)
*Pediococcus acidilactici*	Broth medium	Gündodu et al. (2006)
*Pediococcus pentosaceus*	Raw pork meat batters	Li et al. (2013)
*Pediococcus pentosaceus SWU73571*	Sour meat	Zhang et al. (2020b)
*Pediococcus pentosus CMRC 7*	Guizhou Fermented Meat （Nanx Wudl）	Chen et al. (2018)
*Chromobacterium violaceum Kurthiasp*	Horse heart myoglobin	(Alahakoon et al., 2015; Arihara et al., 1993)
*Complex strains PRO-MIX5(Staphylococcus xylose + Lactobacillus sakei + Lactobactillus plantarum)*	Bacon	Liu et al. (2021)

### 7.3 Cooking method

Nitrite is easily oxidized in high-temperature aerobic environments, such as cooking. Therefore, cooking can decrease the nitrite content of meat or meat products. Nitrate reduction and nitrite accumulation were observed in the uncooked sausage models, but Waga et al. (2017) found that nitrate inhibits nitrite auto-decomposition in pork ham cooked in a water bath at 75 °C. Asioli et al. (2017) investigated the effects of dry-frying temperatures (100, 150, 200, and 250 °C) on the residual nitrite and N-nitrosamine levels in smoked bacon, and found that the residual nitrite content initially increased (from unheated control to 150 °C) and then sharply decreased between 150 °C and 250 °C. Sallan et al. (2020) investigated the effects of cooking extent on volatile nitrosamine formation in the heat-treated semi-dry fermented sausage sucuk, and found that higher temperature and higher heat intensity induced more nitrite controverted into nitrosamines, the content of nitrite and cooking levels influenced the content of N-nitrosopiperidine (NPIP) more than other nitrosamines, well done and very well done cooking levels combining with higher nitrite contents (100 and 150 mg/kg) resulted in significant increases in NPIP content. Recent investigations into the effects of cooking in this context recommend it for the reduction of residual nitrite in meat and meat products.

### 7.4 Irradiation

Food irradiation includes treatment with gamma rays, X-rays, or electron beams. Among these methods, gamma irradiation has the highest potential. However, its development and commercialization have been hampered by unfavorable public perceptions. The endorsement of food irradiation by many international food and health organizations has counteracted this perception to some degree; thereby increasing consumers’ confidence and raising food industry interest. Gamma irradiation is effective in reducing the residual nitrite and N-nitrosamines in meat products. A model sausage that was irradiated with γ-irradiation found that high dose irradiation (>10 kGy) could decrease the residual nitrite levels significantly (Ahn et al., 2002). A dose of 5 kGy γ-irradiation significantly decreased residual nitrite in Chinese Rugao ham before ripening (Wei et al., 2009). In recent years, limited investigations on effect of irradiation on reduction of the residual nitrite were reported.

### 7.5 Plasma-treated water

The interaction of plasma with water can result in the generation of nitrate and nitrite, as well as reactive oxygen species (Chen et al., 2021; Moutiq et al., 2020). Therefore, plasma-treated water can be used as a substitute for nitrite or nitrate. Moutiq et al. (2020) used atmospheric cold plasma to treat chicken breast meat and found that levels of natural microflora were approximately 2 log CFU/g lower following treatment with 100 kV atmospheric cold plasma for 5 min, and that the shelf life of the chicken breast was extended by sterilization of the mesophiles, psychrotrophs, and *Enterobacteriaceae* in the meat. Dong and Tu (2006) used atmospheric nonthermal plasma to treat roasted lamb, and found that 45 min plasma treatment resulted in 30% lower residual nitrite compared with the lamb treated with added nitrite, while the overall sensory scores were similar in both groups.

### 7.6 Other technologies

As the food industry develops, clean labels are widely preferred and demanded by consumers. Therefore, natural food additives, non-destructive technologies, and minimally harmful processing technologies will be developed to reduce or replace the addition of nitrite or nitrate (Ferysiuk and Wojciak, 2020; Fraqueza et al., 2018; Ranasinghe, 2018; Zhang et al., 2016; Zhang et al., 2020a). In terms of naturally sourced additives, there are more than 2000 edible vegetables, extracts of most of which remain to be evaluated for nitrite reduction. Among non-destructive technologies, ohmic heating and its combination with other non-destructive technologies have not yet been investigated. Minimally harmful processing technologies with potential include bio-preservation methods, bacteriocinogenic cultures, and genetically modified fermentation strains, all of which need to be investigated.

The advantages and disadvantages of ten alternative methods are listed in Table 6. Among them, the use of alternative additives, bacterial fermentation, improved packaging, and high hydrostatic pressure methods have all been frequently investigated in recent years; the replacement of nitrite and nitrate with vegetable extracts has been industrialized, and some natural additives have been commercialized. Meat quality improvement methods have been evaluated and tried in meat production, but the resulting products are expensive and are not accepted by most consumers. Cooking and storage condition methods are used in food processing and storage. Irradiation, plasma-treated water, and UV light methods are emerging technologies, and their main function is to sterilize spoilage bacteria and pathogens in meat or meat products. However, their effects on color, flavor, and oxidation of meat or meat products remain to be further investigated.

**Table 6:** Advantages and disadvantages of the alternative methods.

Alternative methods	Advantages	Disadvantages
Meat quality improvement	Reduce or partially replace nitrite or nitrate, improve the flavor of meat	Storage intolerance, high feeding cost, the meat price is high
Alternative additives	Reduce or partially replace nitrite or nitrate, enrich nutrients of meat products, improve the flavor of meat products	Effect is not as good as adding nitrite, need a variety of additives, increased safety control risk,
Bacteria fermentation	Reduce or partially replace nitrite or nitrate, produce unique flavor, extend shelf life, improve product texture and protein absorption rate; reduce the pH and moisture content	The production process is complex, there is the risk of harmful bacteria pollution, the production cost is high, some products tastes sour
Cooking	Reduce or partially replace nitrite or nitrate, saves cost, it can make the product produce good flavor, the cooked meat or meat products are convenient to eat	It is mainly applicable to ready to eat products, long-term steaming will lead to the increase of nitrite content in sausage and cured meat, require heat energy
Storage conditions	Reduce the residual nitrite content, low operation cost, easy to implement, it is not easy to cause deterioration of meat or meat products	Sensory quality of meat or meat products will be influenced, it takes a long time, prolonged exposure to oxygen can easily lead to fat or protein oxidation
High hydrostatic pressure	Reduce or partially replace nitrite or nitrate, it can kill microorganisms, reduce nutrient loss caused by heat treatment, it can be used to process food materials with new functional characteristics, it can be carried out at room temperature or even lower, the processing procedure is simple; there are no strict requirements on the size and shape of meat or meat products	High equipment cost, not applicable to the processing of large meat products, the meat products should be vacuum packed before processing, ultra high pressure process requires intermittent operation, there is a risk of cross contamination between pressure medium and meat and meat products
Irradiation	Reduce or partially replace nitrite or nitrate, it can prevent and control food-borne pathogenic bacteria and spoilage bacteria and keep the quality of meat products	Long time irradiation will produce peculiar smell, not suitable for raw meat and its products, it will cause the loss of nutrients to a certain extent, it is harmful to human body and requires special protection
Plasma-treated water	Reduce or partially replace nitrite or nitrate, fast sterilization speed, high sterilization efficiency, pollution-free, efficient and environmental protection	It is suitable for sterilization of unpacked meat and meat products, for surface sterilization only, it damage protein structure
Package method	Reduce or partially replace nitrite or nitrate, It can maintain the quality, color, flavor and nutrition of meat and meat products, It can prolong the shelf life of meat and meat products	It is not suitable for irregular bone meat and its products, vacuum packaging can lead to the accumulation of juice on the surface of meat products, packaging materials pollute the environment
Illumination method	Reduce or partially replace nitrite or nitrate, high sterilization efficiency and simple equipment, no toxic and harmful substances will be produced, it has a certain odor removal effect, the equipment covers a small area and is easy to maintain	It is only applicable to the surface sterilization of meat and meat products, there is no continuous disinfection ability, there may be a problem of microbial photoreactivation, it cannot sterilize spores, cysts and viruses

## 8 Conclusions

Nitrite is a vital additive in meat and meat products. To date, the multiple functions of nitrite in meat processing cannot be completely replaced by other additives. To ensure that the use of nitrite or its oxide nitrate is safe for humans, regulations and directives have been adopted by various countries and regions, and 6 alternative methods of reducing or partially replacing nitrite in meat processing have been described in this review. Among these methods, alternative additives may be the most successful methods of replacing nitrite in meat processing, because there were many recently investigations focused on this direction. The analysis and comparison of recent investigations into nitrite and nitrate in meat and meat products have found that there are many unanswered questions that require further investigation. We suggest that the correlation between the risk of cancer and consumption of cured meat should be systematically researched. In particular, the main contributors to CRC among vegetables, water, red meat, and meat products should be clarified. The sampling survey by regulators should pay more attention to raw and fried meat products. Ohmic heating and other alternative methods or additives, which can improve the cleanliness of labels and are conducive to the production of high-quality meat or meat products, should be further studied and developed.

## CRediT authorship contribution statement

**Yin Zhang:** Conceptualization, Writing – review & editing. **Yingjie Zhang:** Methodology. **Jianlin Jia:** Methodology. **Haichuan Peng:** Investigation. **Qin Qian:** Investigation. **Zhongli Pan:** Review, review and revise the lanaguage of this review. **Dayu Liu:** Investigation.

## Declaration of competing interest

The authors declare that they have no known competing financial interests or personal relationships that could have appeared to influence the work reported in this paper.

## Data Availability

Data will be made available on request.
